# *QuickStats:* Percentage* of Adults in Fair or Poor Health,^^†^^ by Age Group and Race and Ethnicity^^§^^ — National Health Interview Survey, United States, 2019

**DOI:** 10.15585/mmwr.mm7009a5

**Published:** 2021-03-05

**Figure:**
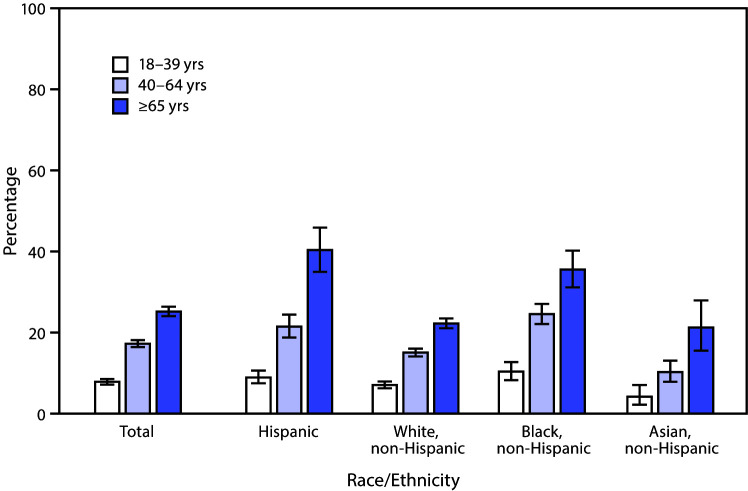
In 2019, the percentage of adults in fair or poor health increased by age (7.8% for those aged 18–39 years, 17.2% for those 40–64 years, and 25.1% for those ≥65 years) and for each racial/ethnic group shown. Hispanic and non-Hispanic Black adults were most likely to be in fair or poor health in each age group. Among persons aged 18–39 and 40–64 years, non-Hispanic Asian adults were least likely to be in fair or poor health. Among persons aged ≥65 years, non-Hispanic Asian and non-Hispanic White adults were least likely to be in fair or poor health. Hispanic and non-Hispanic Black adults aged ≥65 years had the highest percentages of fair or poor health (40.3% and 35.5%, respectively), and non-Hispanic Asian adults aged 18–39 years had the lowest percentage of fair or poor health (4.1%).

**Source:** National Center for Health Statistics, National Health Interview Survey, 2019 data. https://www.cdc.gov/nchs/nhis.htm

